# High expression of centromere protein N as novel biomarkers for gastric adenocarcinoma

**DOI:** 10.1002/cnr2.1798

**Published:** 2023-03-14

**Authors:** Xiaojie Wang, Keyuan Zhang, Cun Fu, Fei Wu, Junjie Zhang, Bin Han, Hai Pan, Lan Luan

**Affiliations:** ^1^ Department of Pathology Central Hospital Affiliated to Shenyang Medical College Shenyang China; ^2^ Basic Medical School Shenyang Medical College Shenyang China; ^3^ Department of Urology Shengjing Hospital of China Medical University Shenyang China; ^4^ Central Laboratory Central Hospital Affiliated to Shenyang Medical College Shenyang China

**Keywords:** apoptosis, CENPN, cycle, gastric adenocarcinoma, proliferation

## Abstract

**Background:**

The role and mechanism of centromeric protein N (CENP‐N), which has been associated with the development of various cancer types, are yet unclear in stomach adenocarcinoma (STAD).

**Methods:**

Data from the Cancer Genome Atlas and Genotype‐Tissue Expression were used to determine whether CENP‐N expression was altered in STAD tumors compared to normal tissues. Xiantao was used to perform Gene Ontology (GO) and Kyoto Encyclopedia of Genes and Genomes(KEGG) enrichment analysis on CENP‐N. The relationship between CENP‐N expression and immune cell infiltration was assessed using TCGA database. The expression of CENP‐N in STAD and surrounding tissues was confirmed using immunohistochemical staining and the correlation between CENP‐N expression and clinicopathological characteristics was examined. The effects of CENP‐N knockdown by siRNA on proliferation were measured by CCK‐8 and EdU assays in AGS cells. Following siRNA transfection, flow cytometry was performed to evaluate cell cycle and apoptotic alterations in AGS cells. The effect of CENP‐N knockdown on the expression level of related proteins was detected by Westren blot.

**Results:**

CENP‐N was highly expressed in STAD tissues, which was confirmed by our immunohistochemistry results. The degree of invasion, TNM stage, and lymph node metastases were all strongly associated with CENP‐N expression. CENP‐N was essential for the cell cycle, DNA replication, chromosomal segregation, and nuclear division; there was a positive correlation between CENP‐N expression and infiltrating Th2 and NK CD56dim cells and a negative correlation between CENP‐N expression and mast, pDC, NK, and B cell infiltration. When CENP‐N expression in AGS cells was knocked down, cell proliferation dramatically reduced (*p* < .05) and the percentage of cells in the S and G2‐M phases decreased significantly (*p* < .05). Silencing CENP‐N significantly promoted the apoptosis of AGS cells (*p* < .05). Mechanistic investigations showed that silencing CENP‐N expression may inhibit STAD proliferation through the Cyclin E1 and promote STAD apoptosis through the Bcl‐2/Bax.

**Conclusion:**

According to our data, CENP‐N acts as an oncogene in STAD and may be a viable therapeutic target.

## 1 INTRODUCTION

Stomach cancer (STAD) is the third most common cause of cancer‐related death worldwide. The high fatality rate is commonly attributed to late diagnosis where the disease is already at an advanced stage.^1^ Therefore, early identification and diagnosis are critical to improve clinical outcomes. Currently, the methods adopted for the timely diagnosis of STAD have several flaws. For example, tumor marker specificity and sensitivity are insufficient,^2^, ^3^ gastroscopy screening is difficult to implement, and imaging examination plays a limited role in the identification of early STAD.^4^ Therefore, the discovery of novel biomarkers with high specificity and sensitivity is critical.

The centromere is a region of specialized chromatin, which is comprised of a combination of DNA and proteins. Although centromeric DNA sequences differ greatly among species, their protein components are relatively conserved.^5^, ^6^ These highly conserved proteins are called centromere‐associated proteins (CENPs).^7^ According to previous studies, CENPs control the normal separation of sister chromatids and maintain chromosome stability, making them an important factor in the occurrence and progression of tumors.^7^, ^8^, ^9^, ^10^ CENPN is an important member of the centromeric protein family.^11^ In the constitutive centromere associated network (CCAN), CENP‐N and CENP‐L could form the CENP‐L/N complex, which could recruit other centromere proteins to the centromere by identifying CENP‐A nucleosomes. This was essential for CCAN protein assembly and accurate chromosome separation.^12^ Studies have shown that CENP‐N is highly expressed in diverse carcinomas including gliomas, liver cancer, and lung cancer with roles in tumorigenesis, cell proliferation, and apoptosis.^13^, ^14^, ^15^ However, the role of CENP‐N in gastric adenocarcinoma remains unclear.

In this study, we investigated the expression of CENP‐N in STAD and its effects on proliferation, cell cycle progression, and apoptosis in a gastric adenocarcinoma cell line (AGS) using bioinformatics, immunohistochemistry (IHC), and functional experiments.

## 2 MATERIALS AND METHODS

### 2.1 Patients and specimens

The study included 76 wax block specimens surgically resected from confirmed patients with STAD at the Department of Pathology, Affiliated Central Hospital of Shenyang Medical College, between June 2014 and July 2016. Healthy tissue samples from the same patients were used as the control group. The study included 53 males and 23 females with an age range of 36 to 95 years and a mean age of 66 years. Included patients had not received radiotherapy or chemotherapy before surgery. This study was approved by the Medical Ethics Committee of the Shenyang Medical College, China.

### 2.2 Data gathering and analysis

The Gene Expression Profiling Interactive Analysis (GEPIA) database was used to analyze the expression of CENP‐N in STAD sample data from The Cancer Genome Atlas (TCGA) and The Genotype‐Tissue Expression (GTEx) databases. The conditions for analysis were as follows: | Log2FC | Cutoff: 1, *p*‐value cutoff: .01, matched Normal data: match TCGA normal and GTEx data, containing 408 gastric adenocarcinoma tissue samples and 211 normal control tissue samples. Using the Xiantao academic database, the expression of CENPN in TCGA‐STAD was analyzed in tumor and normal tissues. ROC curve was analyzed by the TCGA‐GTEx‐STAD data set.

### 2.3 CENP‐N functional enrichment analysis and screening of co‐expressed genes in STAD

TCGA‐STAD data were used to perform a Pearson correlation study in CENP‐N and other mRNAs in gastric cancer. Enrichment analysis was used to assess the function of CENP‐N. The 300 genes with the highest positive correlation values with CENP‐N were chosen. Gene ontology (GO) and Kyoto encyclopedia of genes and genomes (KEGG) pathway analyses were performed using Xiantao Academic.

### 2.4 Immune infiltration analysis

The Xiantao Academic and single‐sample gene set enrichment analysis were used to examine the association between CENP‐N expression in STAD and 24 different types of immune cells (ssGSEA). Additionally, the relationship between CENP‐N expression and Th2, NK CD56dim cells, mast cells, pDC, NK cells, and B cells was examined using Spearman's correlation.

### 2.5 Immunohistochemical staining

After roasting, dewaxing, antigen repair, and endogenous peroxidase blocker, paraffin sections were incubated with rabbit polyclonal anti‐CENP‐N antibody (1400 dilution, Proteintech, Rosemont, IL) and stored in a refrigerator at 4°C overnight. The next day, the sections were left at 25°C for 30 min, followed by incubation with the secondary antibody (Fuzhou Maixin Biotechnology Development Co. Ltd, Fuzhou, China) at 25°C. Sections were then stained with DAB and hematoxylin, dehydrated with absolute alcohol for 10 min, and sealed with neutral gum.

Evaluating outcomes: The cytoplasm and nucleus were the main sites for CENPN expression. Two pathologists performed a blind review of the immunohistochemical results. Five visual fields were chosen at random, and the staining intensity and percentage were assessed and assigned a semi‐quantitative score. The percentage scores given for stained cells were 0 (4% or less), 1 (5%–24%), 2 (25%–49%), 3 (50%–74%), or 4 (75%). The staining intensities were assigned 0 (no staining), 1 (light yellow particles), 2 (yellow), and 3. (dark yellow particles). The staining intensity score was multiplied by the percentage score for positively stained cells in each tumor sample to yield a total score ranging from 0 to 12. A score of less than 3 was considered negative, and a score of 3 or higher was considered positive.

### 2.6 Cell culture and transfection

AGS cells were obtained from the Shanghai Cell Bank of the Chinese Academy of Sciences (Shanghai, China) and were routinely sub‐cultured in F12 medium (Gibco, Thermo Fisher, Waltham, MA) containing 10% fetal bovine serum, penicillin (100 U/mL), and streptomycin (100 mg/mL) at 37°C in a cell incubator perfused with 5% CO_2_.

The negative control, siRNA‐NC, and si‐CENP‐N were purchased from RiboBio Co. Ltd. (Guangzhou, China). The sequences of CENP‐N siRNA were as follows: siRNA1:5’‐CTACCTACGTGGTGTACTA‐3′; siRNA2: 5’‐GTGATGCTGCCCTGTTAGA‐3′; siRNA3: 5’‐CATGCTGAGGCGCAATACA‐3′. Transfection of siRNAs was carried out in accordance with the manufacturer's instructions using Lipo3000 reagent (Life Technology Company, Carlsbad, CA). After 48 h of transfection, the depletion of CENP‐N protein was examined using western blotting in gastric cancer cells that were in the logarithmic growth phase.

### 2.7 Western blot analysis

Cells were lysed using RIPA buffer according to the manufacturer's protocol. Protein (30 μg/lane) was added to the 10% SDS‐PAGE gel and then transferred to a PVDF membrane. The membrane was blocked with 5% skim milk for 1 h in TBST and incubated with primary antibodies (Bcl‐2, Bax, p53 and CDK2 purchased from Proteintech, Rosemont, IL; CyclinE1 purchased from ZEN‐BIOSCIENCE, Durham, NC) at 4°C overnight. Membranes were then washed thrice and incubated with secondary antibody (Maixin Biotechnology Development Co, Ltd, Fuzhou, China) for 1 h. The signal was detected using an enhanced chemiluminescence western blot assay kit.

### 2.8 CCK‐8 assay

In a 96‐well plate, 5 × 10^3^ cells were seeded into each well. To each well, 10 μL of CCK‐8 (APExBio, Houston, TX) was added at 0, 24, 48, 72, and 96 h. A microplate reader (TECAN Infinite M200pro, Zürich, Switzerland) was used to measure the optical density at 450 nm following 1 h incubation at 37°C, and a cell growth curve was determined.

### 2.9 EdU cell proliferation assay

Cells were inoculated with 5 × 10^4^ cells per well in a 12‐well plate. Transfection was performed accordingly. Each well was labeled with diluted EdU (1:5000 dilution; Ruibo Biotechnology Co., Ltd., Guangzhou, China) and incubated for 48 h at 37°C in a 5% CO_2_ incubator. The cells were fixed, penetrant was added, and they were then stained. Hoechst 33 342 was then added to each well at a dilution of 1:1000. After 15 min of shaking at room temperature (17–21°C) in the dark, the dye solution was removed, and the cells were washed with phosphate‐buffered saline. Finally, an inverted microscope was used for observation where five different photographic fields were picked at random.

### 2.10 Cell cycle detection

Cell cycle progression was detected using a cell cycle detection kit (BD Pharmingen, San Diego, CA) according to the manufacturer's instructions. Cells in 6‐well plates were transfected for 48 h, fixed in 75% ethanol, labeled with propidium iodide, and analyzed using flow cytometry (BD FACSCalibur, Marshall Scientific, Hampton, NH). Each group consisted of three replicates. Data was analyzed using Modfit software.

### 2.11 Detection of cell apoptosis

Apoptosis was measured using an apoptosis detection kit (Vazyme, Nanjing, China). Cells were transfected for 48 h in 6‐well plates, stained with annexin V and propidium iodide, and analyzed using flow cytometry (BD FACSCalibur, Marshall Scientific, Hampton, NH). Data was analyzed using Cellquest software.

### 2.12 Statistical analysis

Statistical analysis was performed using Statistical Product and Service Solutions (SPSS) Statistics version 20.0 (IBM Corp., Armonk, NY, USA) and GraphPad Prism 9 (GraphPad Software, La Jolla, CA, USA).Every experiment included at least three independent replicates. For immunohistochemistry (IHC) samples, the correlation of CENPN expression and clinicopathological features was tested using a χ^2^ test. Data comparing CENPNsiRNA and siNC were tested using a *t*‐test. *p* < .05 was considered significant. Spearman's correlation analysis was used for correlation analysis.

## 3 RESULTS

### 3.1 Expression of CENP‐N in STAD tissue

**FIGURE 1:**
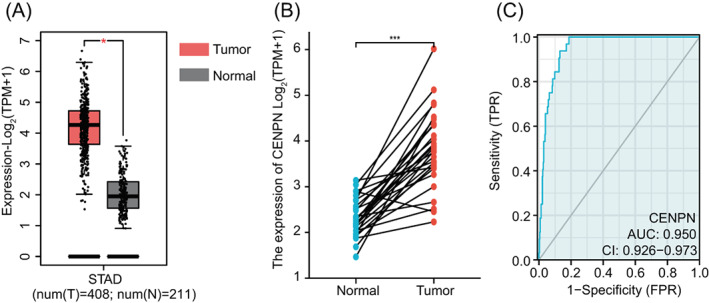
CENP‐N expression analysis in STAD. (A) CENP‐N expression in tumor and normal tissues based on STAD data from TCGA and GTEx. (B) CENP‐N expression in paired tumor and normal tissues in STAD from TCGA. (C) ROC curves for classifying gastric adenocarcinoma versus normal gastric tissues in the TCGA database. TCGA, The Cancer Genome Atlas; GTEx, Genotype Tissue Expression Project; ROC, receiver operating characteristic. Data are shown as mean ± SD. *p < .05, ***p < .001.

Using the GEPIA database, we first examined the expression of CENP‐N in STAD data from the TCGA and GTEx databases. STAD tissues had significantly higher levels of CENP‐N mRNA expression as compared to healthy neighboring tissues (Figure 1A, *p* < .05). We used the TCGA database to assess the expression of CENP‐N in STAD paired samples; the expression of CENP‐N in STAD was higher than that in matched normal tissues in 27 samples (Figure 1B, *p* < .001). In addition, the ROC curve showed that CENP‐N expression had a good predictive ability, with an area of 0.950 under the curve (95% confidence interval [CI] = 0.926–0.973), and STAD tissues could be distinguished from normal tissues (Figure 1C).

**FIGURE 2:**
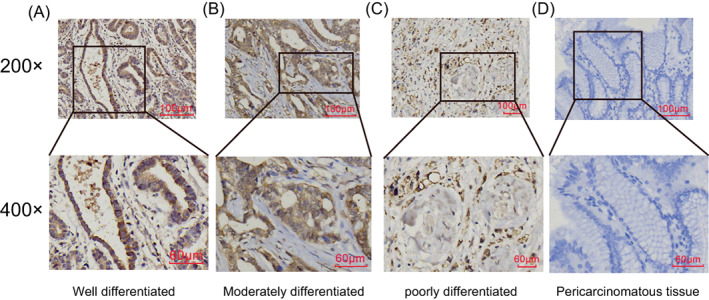
The results of Immunohistochemistry. (A–D) Representative images of CENP‐N expression in gastric adenocarcinoma tissues and their matched normal tissues. Scale bars represent 100 μm (200×) and 60 μm (400×).

To further validate CENP‐N expression in STAD, IHC staining was performed on 76 tumor specimens and adjacent healthy tissues to assess the differential expression of CENP‐N. CENP‐N was highly expressed in STAD tissues, with low or no expression in control tissue samples. The highest expression was detected in the cytoplasm and nucleus (Figure 2A–D). The positivity rate in STAD tissues was 76.3%, whereas it was 18.4% in adjacent tissues (*χ* ^*2*^ = 51.089, *p* = .019) (Table 1).

**TABLE 1:** CENP‐N expression in STAD and adjacent healthy tissues.

Tissue type	Number of patients	CENPN		χ^2^ value	*p* value
		Negative (%)	Positive (%)		
Gastric carcinoma	76	18 (23.7)	58 (76.3)	51.089	<.01
Paracancerous tissue	76	62 (81.6)	14 (18.4)		

### 3.2 Relationship between CENP‐N expression in STAD and clinicopathological parameters

We investigated the relationship between CENP‐N expression and various clinicopathological parameters in patients with STAD to better understand the significance and possible molecular mechanisms underlying CENP‐N expression in the development of STAD. The expression of CENP‐N was significantly different in STAD patients with varying degrees of invasion (*χ* ^*2*^ = 8.507, *p* = .004), TNM stage (*χ* ^*2*^ = 7.991, *p* = .005), and lymph node metastasis (*χ* ^*2*^ = 4.353, *p* = .037). However, there was no statistically significant difference in CENP‐N expression according to sex, age, tumor size, or degree of differentiation (Table 2). Interestingly, however, we found that TNM stage was positively correlated with patient age and tumor size, while the depth of invasion and nodal status were correlated with patient tumor size (Table 3).

**TABLE 2:** The association of CENP‐N expression with clinicopathological characteristics in STAD.

Characteristics	Number of patients	CENP‐N		χ^2^ value	*p* value
		Negative (%)	Positive (%)		
Gender				0.723	.395
Male	53	14 (26.4)	39 (73.6)		
Female	23	4 (17.4)	19 (82.6)		
Age				0.158	.691
<65 (years)	24	5 (20.8)	19 (79.2)		
≥65 (years)	52	13 (25.0)	39 (75.0)		
Tumor size (cm)				1.653	.198
<6	45	13 (28.9)	32 (71.1)		
≥6	31	5 (16.1)	26 (83.9)		
Differentiation				0.487	.485
Well‐moderate	41	11 (26.8)	30 (73.2)		
Poor	35	7 (20.0)	28 (80.0)		
Depth of invasion				8.507	**.004**
T1 + T2	25	11 (44.0)	14 (56.0)		
T3 + T4	51	7 (13.7)	44 (86.3)		
TNM stage				7.991	**.005**
I + II	37	14 (37.8)	23 (62.2)		
III + IV	39	4 (10.3)	35 (89.7)		
Nodal status				4.353	**.037**
N0	23	9 (39.1)	14 (60.9)		
N1 N2 N3	53	9 (17.0)	44 (83.0)		

**TABLE 3:** Correlation analysis of pathological factors.

Variable	Gender		Age	Tumor size(cm)		
	*R* value	*p* value	*R* value	*p* value	*R* value	*p* value
Depth of invasion	0.201	.081	−0.004	.972	0.249	**.03**
TNM stage	0.116	.317	0.26	**.023**	0.32	**.005**
Nodal status	0.004	.976	0.042	.721	0.281	**.014**

### 3.3 CENP‐N functional enrichment analysis and co‐expression gene screening in STAD

**FIGURE 3:**
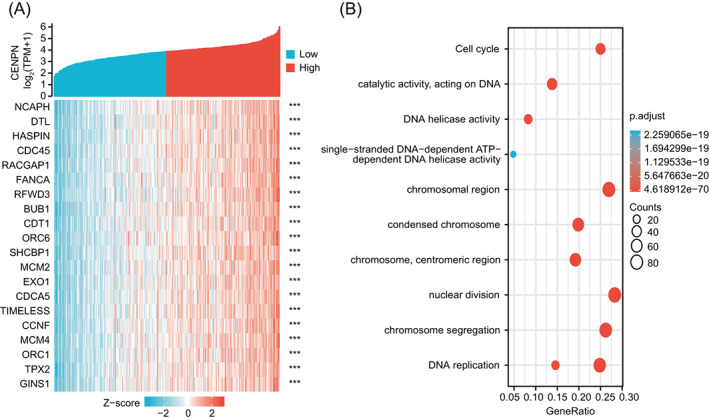
STAD co‐expression gene screening and functional enrichment analysis of CENP‐N. (A) Heat map displaying the 20 genes that had the strongest positive association with CENP‐N expression. (B) GO|KEGG pathway analysis of the 300 genes that had the strongest positive association with CENP‐N. Data are shown as mean ± SD. ***p < .001.

To further investigate the functions and pathways affected by CENP‐N, we used TCGA data to examine the correlation between CENP‐N and other genes associated with STAD. The top 300 genes most strongly associated with CENP‐N were chosen for enrichment analysis, and the top 20 genes are displayed in a heat map (Figure 3A). Using the Xiantao database, we examined potential functional pathways based on the top 300 genes. GO and KEGG enrichment analysis revealed that CENP‐N plays important roles in the cell cycle, DNA replication, chromosome separation, and nuclear division, as well as several other key signaling pathways (Figure 3B).

### 3.4 Correlation between immune cell infiltration and CENP‐N expression

**FIGURE 4:**
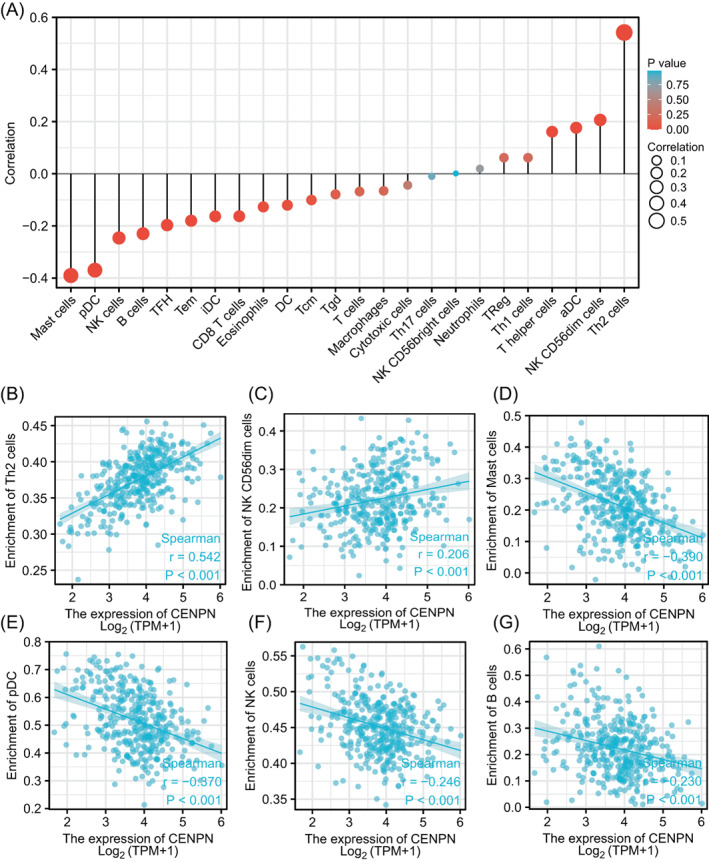
Correlation of CENP‐N expression with immune infiltration level in STAD. (A) Correlation between CENP‐N expression and relative abundance of 24 types of immune cell. The size of each dot corresponds to the absolute Spearman's correlation coefficient values. (B–G) Correlation of CENP‐N expression with infiltration levels of Th2, NK CD56dim, mast, pDC, NK, and B cells in STAD. Th2 cells, T‐helper type 2 (Th2) cells; NK CD56dim cells, Natural killer CD56dim cells; pDC cells, plasmacytoid dendritic cells; NK cells, natural killer cells.

Immune cells that infiltrate tumors play a significant role in the tumor microenvironment, and this infiltration is linked to the development, spread, and metastasis of cancer.^16^, ^17^ Therefore, we examined the connection between CENP‐N expression in STAD and the degree of immune cell infiltration (Figure 4A). A positive correlation was determined between CENP‐N expression and Th2 and NK CD56dim cell populations (Figure 4B,C). Mast, pDC, NK, and B cell populations were negatively correlated with the expression of CENP‐N (Figure 4D–G).

### 3.5 Effect of CENP‐N on AGS cell proliferation

**FIGURE 5:**
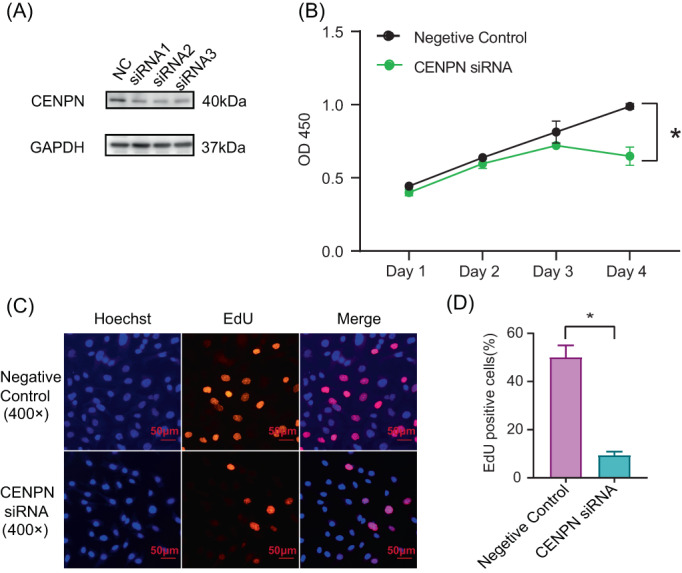
Effects of CENP‐N knockdown on the proliferation of STAD cells. (A) Expression of CENP‐N detected after transfection with siRNA using western blot. (B) Representative images of CCK‐8 assay. (C) Representative images of EdU assay as quantified in (D). Scale bars represent 50 μm (400×) Data are shown as mean ± SD. *p < .05.

To better understand the regulatory function of CENP‐N on the proliferation of STAD cells, we transiently transfected siRNAs to downregulate CENP‐N expression in AGS cell lines. The transfection effect was confirmed using western blotting. The CENP‐N expression in AGS cells in the transfection group was significantly lower than that in the control group (Figure 5A). We evaluated the proliferation and growth of AGS cells after CENP‐N downregulation using the CCK‐8 assay. The results showed that CENP‐N downregulation significantly reduced the proliferation of AGS cells compared to the control group (Figure 5B, *p* < .05). To determine the effect of CENP‐N on STAD cell proliferation, an EdU test was also performed. The findings demonstrated that CENP‐N siRNA‐transfected AGS cells had considerably lower proliferation ratios than the control group (Figure 5C,D, *p* < .05). In conclusion, the downregulation of CENP‐N may inhibit the proliferation of STAD cells.

### 3.6 Effect of CENP‐N on the cell cycle of AGS cells

**FIGURE 6:**
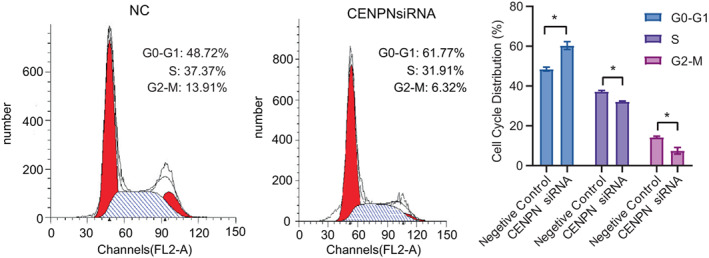
Flow cytometry assays were conducted to analyze the effect of CENP‐N downregulation on the cell cycle in AGS cells. Data are shown as mean ± SD. *p < .05.

To further investigate the mechanism by which CENP‐N regulates the proliferation of human AGS cells, flow cytometry was used to detect cell cycle changes. The CENP‐N knockdown in AGS cells resulted in an increased percentage of G0‐G1 cells compared to the controls, while the percentage of cells in the S and G2‐M phases decreased significantly (Figure 6, *p* < .05). These findings imply that CENP‐N may promote cell proliferation by regulating the cell cycle.

### 3.7 Effect of CENP‐N on AGS cell apoptosis

**FIGURE 7:**
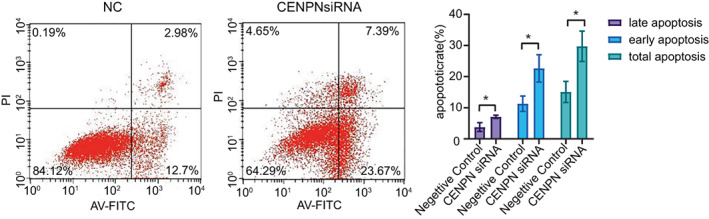
Flow cytometry assays were conducted to analyze the effect of CENP‐N downregulation on cell apoptosis in AGS cells. Data are shown as mean ± SD. *p < .05.

We used flow cytometry to investigate the role of CENPN in cell apoptosis in STAD. The percentages of early, late, and complete apoptosis in AGS cells were 23.67% (12.7% in the control group), 7.39% (2.98% in the control group), and 31.06% (15.68% in the control group), respectively. After CENPN knockdown, the apoptosis rate of AGS cells was considerably higher than that of the control group (Figure 7, *p* < .05). Therefore, we conclude that CENPN knockdown promotes apoptosis in STAD cells.

### 3.8 CENP‐N acts in the AGS cell via Cyclin E1 and Bcl‐2/Bax

**FIGURE 8:**
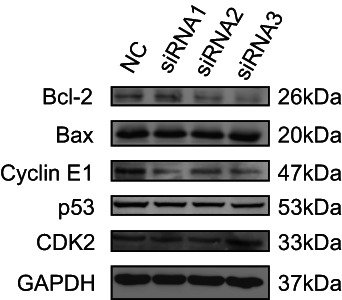
The effect of CENPN expression on the expression of related proteins was analyzed by Western blotting.

To elucidate the mechanism through which CENP‐N expression promotes STAD development, we studied the effects of CENP‐N on several oncogenic signaling pathways using western blotting. The results showed that the Cyclin E1 expression level and Bcl‐2/Bax ratio were decreased in the CENP‐N knockdown group as compared to the control. There was no significant difference in expression levels of p53 and CDK2 (Figure 8). Therefore, we suggest that CENP‐N knockdown inhibits STAD proliferation through the Cyclin E1 pathway and promotes STAD apoptosis through the Bcl‐2/Bax pathway.

## 4 DISCUSSION

CENP‐N encoded proteins form nucleosome‐associated complexes that are important for centromere assembly. CENP‐N binds to centromeres during the S and G2 phases of the cell cycle and recruits other centromeric proteins.^18^ Accurate chromosome segregation during mitosis is required for the proper transfer of genetic material from the mother to daughter cells. Missegregation of chromosomes can result in aneuploidy, a hallmark of cancer.^8^ In humans, the histone H3 variant centromere protein A (CENP‐A) is present in the functional centromeres. CENP‐A disruption or mutation results in failure of centromere formation.^19^, ^20^ CENP‐N can recognize CENP‐A and regulate mitosis by forming the CENP‐A nucleosome‐associated complex (NAC).^12^, ^21^ CENP‐N interacts with the centromere‐targeting domain of CENP‐A to facilitate centromere assembly,^11^ and CENP‐N depletion leads to the mislocalization of many other CENPs.^21^, ^22^ Consequently, the CENP‐A nucleosome core region, which is necessary for centromere formation and chromosome segregation, is directly recognized by CENP‐N.^23^ However, despite a potentially highly significant role in STAD etiology, the mechanism of CENP‐N in STAD has not yet been reported.

TCGA and GTEx datasets were used in this study to evaluate the expression of CENP‐N in STAD. The results showed that STAD tissues had higher CENP‐N expression levels than healthy adjacent tissues (*p* < .05). We also examined CENP‐N expression in STAD‐paired samples from the TCGA database. The results showed that STAD tissues had a greater level of CENP‐N expression than matched normal tissues (*p* < .001). We verified this finding using IHC. These results are consistent with the high expression of CENP‐N in malignancies of the lungs, liver, nasopharynx, and mouth.^14^, ^15^, ^24^, ^25^ The ROC curve analysis also supports CENP‐N's high diagnostic value. Additionally, we discovered that the degree of tumor invasion, TNM stage, and lymph node metastasis were substantially correlated with the level of CENP‐N expression in the tissues of patients with STAD, indicating that the abnormal expression of CENP‐N was intimately linked to the onset of STAD.

CENP‐N expression was associated with pathways related to cell proliferation such as “cell cycle,” “DNA replication,” “chromosome segregation,” and “nuclear division” in our enrichment analysis. To validate this result, first we knocked down CENP‐N in AGS cells and discovered that depletion of CENP‐N significantly inhibited cell proliferation. Second, flow cytometry was used to detect changes in the cell cycle of AGS cells after CENP‐N knockdown. The results showed that silencing of CENP‐N in AGS cells resulted in an increased percentage of cells in G0‐G1 (*p* < .05) and a significant decrease in the proportion of cells in the S and G2‐M phases (*p* < .05). The S phase is the DNA synthesis phase, which is critical for cell division and proliferation. These findings suggest that CENP‐N downregulation inhibits AGS cell proliferation. Studies have shown that CENP‐N knockdown promoted apoptosis in a variety of cancers.^13^, ^15^ This study hypothesizes that CENP‐N might have the same effect in STAD. Flow cytometry was performed to investigate the relationship between CENP‐N expression and apoptosis in AGS cells. The results revealed that CENP‐N knockdown significantly increased the apoptotic rate of AGS cells. This suggests that CENP‐N is involved in apoptosis in STAD. Cyclin E1 is a key regulator of cell proliferation and plays an important role in the cell cycle.^26^ As a member of the anti‐apoptotic family, Bcl‐2 inhibits apoptosis by binding the pro‐apoptotic proteins Bax and Bak. Overexpression of Bcl‐2 has been detected in a variety of malignant tumor cells.^27^ Western blot analysis showed that CENP‐N silencing decreased Cyclin E1 expression and the Bcl‐2/Bax ratio in AGS cells. Therefore, we suggest that CENP‐N knockdown may inhibit the proliferation of STAD through the Cyclin E1 and promote the apoptosis of STAD through the Bcl‐2/Bax.

Immune cells play a crucial role in controlling the malignant behavior of tumor cells.^28^, ^29^, ^30^ Immune cell infiltration has been linked to the development and spread of cancer.^31^, ^32^ In this study, we explored the association between CENP‐N expression and immune cell infiltration in STAD. We determined that CENP‐N expression had a positive correlation with Th2 and NK CD56dim cell populations and a negative correlation with mast, pDC, NK, and B cell populations. These findings imply that the regulation of tumor immunity is significantly influenced by CENP‐N.

However, this study has certain limitations. First, confounding variables may have skewed the data from open databases. Second, the sample numbers were limited in this study, and more cases are required to validate our observations. Finally, further research is needed to determine the precise role of CENP‐N in STAD.

In conclusion, our investigation showed that CENP‐N was upregulated in STAD tissues and that the degree of tumor invasion, TNM stage, and lymph node metastasis were significantly correlated with CENP‐N expression. The proliferation of STAD cells was inhibited by the depletion of CENP‐N, which also decreased the percentage of cells in the S and G2‐M phases and increased the rate of apoptosis. Immune cell infiltration and CENP‐N expression were significantly correlated. Therefore, we believe that CENP‐N could be a possible therapeutic target for STAD.

## AUTHOR CONTRIBUTIONS

**Xiao jie Wang:** Conceptualization (lead); data curation (lead); investigation (lead); visualization (lead); writing – original draft (lead); writing – review and editing (equal). **Ke yuan Zhang:** Investigation (supporting). **Cun Fu:** Investigation (supporting). **Fei Wu:** Data curation (supporting). **Jun jie Zhang:** Data curation (supporting). **Bin Han:** Supervision (lead). **Hai Pan:** Supervision (equal). **Lan Luan:** Conceptualization (supporting); writing – review and editing (supporting).

## FUNDING INFORMATION

This study was supported by grants from the Shenyang Science and Technology Plan public health R&D special project under grant 21‐172‐9‐18.

## CONFLICT OF INTEREST STATEMENT

The authors declare that they have no competing interests.

## ETHICS STATEMENT

The studies involving human participants were reviewed and approved by the Ethics Committee of Shenyang Medical College. The patients and participants provided their written informed consent to participate in this study.

## Data Availability

Data sharing is not applicable to this article as no new data were created or analyzed in this study.
